# Understanding art and design teachers’ willingness to adopt artificial intelligence in teaching under resource constraints: a mixed-methods study on perceived usefulness, resource readiness, and creativity-related concerns

**DOI:** 10.3389/fpsyg.2026.1854412

**Published:** 2026-09-03

**Authors:** Shu Chen, Xizhen Li, Xiaoting Liu, Jiaxin Wu, Wenhao Cheng

**Affiliations:** 1 School of Art and Design, Hunan Women's University, Changsha, China; 2 Yiyang Vocational and Technical College, Yiyang, China; 3 Hunan Arts and Crafts Vocational University, Yiyang, China; 4 Changsha University, Changsha, Hunan, China

**Keywords:** art and design teachers, attitude, behavioral intention, creative disciplines, educational artificial intelligence, mixed methods, resource readiness, technology acceptance model

## Abstract

**Introduction:**

Artificial intelligence (AI) is increasingly used in higher education, but teachers’ willingness to adopt AI in creative disciplines may not be fully explained by the instrumental logic of the Technology Acceptance Model (TAM). In art and design education, judgments about AI may also involve concerns about originality, skill development, process-based learning, and pedagogical governance. This study examined art and design teachers’ intentions to adopt AI under resource-constrained conditions.

**Methods:**

An explanatory sequential mixed-methods design was used. The quantitative phase included 101 valid questionnaires and tested relationships among perceived ease of use (PEOU), perceived usefulness (PU), resource readiness (RR), attitude (AT), and behavioral intention (BI) using structural equation modeling with 5,000 bootstrap replications. The qualitative phase involved interviews with eight art and design teachers to contextualize the quantitative findings.

**Results:**

Model fit was mixed (CFI = 0.935, TLI = 0.920, SRMR = 0.059, RMSEA = 0.096). Bootstrap results supported PEOU → PU, PU → BI, AT → BI, and RR → PEOU, whereas PU → AT and RR → PU were not supported. PEOU → AT showed a significant negative relationship, contrary to the hypothesized direction. Although RR → AT was positive, its standardized coefficient exceeded 1 and discriminant validity between RR and AT was insufficient; therefore, this path was not substantively interpreted. Interviews indicated that concerns about originality, skill degradation, shortcuts, copyright, academic integrity, and governance may shape how teachers translate functional evaluations into overall attitudes.

**Discussion:**

Several core TAM pathways were supported, but the model was not fully validated. The findings suggest that AI adoption in art and design teaching involves both instrumental judgments and discipline-specific professional, pedagogical, ethical, and resource considerations. Given limitations in model fit and discriminant validity, the results should be regarded as preliminary and context-specific evidence.

## 1 Introduction

In recent years, artificial intelligence (AI) has entered various aspects of higher education, including lesson preparation, feedback, course resource generation, and creative support. Research on teachers’ technology adoption typically relies on the Technology Acceptance Model (TAM) to explain the relationships among perceived usefulness, perceived ease of use, attitude, and behavioral intention. However, in art and design education, AI also involves judgments about originality, foundational skill training, creative processes, authorship responsibility, and curriculum assessment. Therefore, whether the classical adoption logic can be directly applied to creative disciplines remains to be tested within specific teaching contexts.

Art and design education emphasizes studio-based learning, iterative experimentation, critical feedback, and process-oriented judgment. The low barrier to entry and rapid generation capabilities of AI may expand visual exploration, but could also be seen as a shortcut that bypasses ideation, trial-and-error, and skill development. Thus, ease of use does not automatically translate into positive attitudes. To avoid retroactively deriving theory from outcomes, this study maintains the positive relationships of the classic TAM model as *a priori* assumptions and uses interviews to interpret teachers’ understanding of these relationships.

Beyond personal beliefs, AI teaching also depends on external conditions that are accessible and adaptable to curricula. This study operationally defines resource readiness (RR) as teachers’ perceptions of the availability of AI teaching resources and their alignment with curriculum needs, focusing specifically on whether relevant resources can support particular course planning and instructional implementation. This construct differs from general attitudes toward AI, overall judgments of its utility, and intentions to use it.

Based on this, the study builds upon TAM by incorporating resource readiness as an external implementation condition to test eight pre-specified relationships. Subsequently, qualitative interviews are used to interpret the quantitative results, including supportive, non-supportive, reverse, and unstable estimates. An explanatory sequential design is employed to integrate both types of evidence, rather than revising hypotheses after the results emerge.

This study focuses on art and design faculty at universities in central China, aiming to address the following three interrelated questions.

### 1.1 Research questions

- *RQ1*: To what extent are the eight hypothesized relationships among resource readiness, perceived ease of use, perceived usefulness, attitude, and behavioral intention supported in this study’s sample?
- *RQ2*: How do art and design teachers understand the roles of originality, skill development, process-based learning, and teaching governance in shaping their attitudes toward AI in teaching?
- *RQ3*: How do the qualitative themes explain, complement, or challenge the relationship patterns observed in the quantitative phase, particularly the relationships among instrumental beliefs (PEOU and PU), resource readiness, and attitude?

This study does not pre-emptively claim to uncover the boundaries or value conflict mechanisms of TAM, but instead provides contextualized preliminary evidence on AI adoption among creative discipline teachers through an explanatory sequential mixed methods approach, while assessing the potential role of resource readiness as an external implementation condition.

## 2 Literature review

### 2.1 Traditional TAM explanation of teachers’ AI adoption

The Technology Acceptance Model (TAM) identifies perceived usefulness (PU) and perceived ease of use (PEOU) as the core beliefs underlying technology acceptance: PU reflects the extent to which a technology enhances job performance, while PEOU reflects the effort required to use the technology; PEOU can also strengthen PU, and attitudes further influence behavioral intention (Davis, 1989; Davis et al., 1989). TAM2 and TAM3 subsequently incorporated additional variables such as social influence, cognitive instrumentalality, self-efficacy, anxiety, and perceived control, yet retained these fundamental relationships (Venkatesh and Davis, 2000; Venkatesh and Bala, 2008).

Teacher education technology research generally supports these relationships. Teo (2011) found that perceived usefulness (PU), perceived ease of use (PEOU), attitude, and facilitating conditions were related to teachers’ intention to use technology; Scherer et al. (2019) also demonstrated through a meta-analysis of 114 studies that the Technology Acceptance Model (TAM) and its variants could explain teachers’ adoption of digital technologies. Research on generative AI similarly indicates that PU, attitude, self-efficacy, and institutional support jointly influence teachers’ usage intentions (Kong et al., 2024; Hazzan-Bishara et al., 2025). Therefore, TAM is well-suited as a foundational model for examining teachers’ adoption of AI.

However, teachers also assess whether AI alters professional roles, teaching control, and decision-making transparency (Kim and Kim, 2022), as well as whether resources, training, and reliable information are sufficient to support classroom implementation (Hazzan-Bishara et al., 2025). These considerations are particularly prominent in creative education, which emphasizes value judgments and process-based learning. Therefore, this study uses TAM as a testable framework without equating efficiency or ease of use with ultimate acceptance.

### 2.2 Attitude formation in creative education: from instrumental judgment to value judgment

Art and design education emphasizes not only task completion but also creative generation, esthetic judgment, process reflection, studio interaction, and critique of works to foster design thinking and creative practice (Sawyer, 2017; Meyer and Norman, 2020). Therefore, when teachers evaluate AI, they assess not only its usefulness and ease of use, but also whether it aligns with the discipline’s expectations regarding originality, skill, process, and responsibility. In this context, attitudes emerge more as a synthesis of tool evaluation and disciplinary value judgments.

First, generative AI can enhance the speed or novelty of individual output, but it may also reduce the diversity of the overall body of work (Doshi and Hauser, 2024) and lower perceptions of authenticity by diminishing human involvement (Messer, 2024). Therefore, art and design educators’ understanding of “usefulness” extends beyond efficiency to include whether AI supports recognizable, interpretable, and defensible creative expression (Ivcevic and Grandinetti, 2024).

Second, studio teaching emphasizes observation, material practice, critical feedback, and iterative revision, rather than merely pursuing efficiency in producing finished works (Sawyer, 2017). As AI makes image generation, conceptual development, or design decision-making more convenient, educators may simultaneously worry about students receiving less foundational training and developing weaker judgment. Art and design students’ concerns regarding originality and stylistic homogenization also indicate that ease of use does not automatically equate to pedagogical appropriateness (Shen et al., 2025).

Once again, the value of creative learning lies in the entire process—from problem definition and sketch exploration to feedback and iteration. Interaction, consultation, and peer exchange within a studio environment are essential conditions for design-based learning (Iranmanesh and Onur, 2022); AI involvement may also affect assessment by weakening authorship and human agency in the process (Messer, 2024). Teachers therefore examine whether students’ choices, revisions, and judgments remain visible.

Finally, ethical concerns such as authorship attribution, the legality of training data, fairness in evaluation, academic integrity, and overreliance mean that AI adoption goes beyond mere efficiency considerations (Zawacki-Richter et al., 2019). Whether teachers accept AI also depends on professional competence, institutional support, and pedagogical appropriateness (Nevarez Montes and Elizondo-Garcia, 2025; Zhu et al., 2026), while authentic assessment emphasizes making judgments, revisions, accountability, and ethical choices visible (Kickbusch et al., 2025).

In summary, originality, skill development, process authenticity, and teaching ethics may weaken or reconfigure the classical transformation relationships among PEOU, PU, and AT. This study treats these factors as contextual mechanisms to be explored through interpretive interviews, rather than presupposing them as already established moderating variables or theoretical boundaries.

### 2.3 Resource readiness as an external implementation condition

UTAUT defines facilitating conditions as individuals’ perceptions of the extent to which organizational and technological infrastructure supports their use (Venkatesh et al., 2003). This study’s Resource Readiness (RR) is related to this concept but has a narrower operational scope: it refers specifically to teachers’ perceptions of the availability and curriculum alignment of AI teaching resources, focusing on whether these resources can support specific course planning and instructional implementation. RR is not an objective inventory of school resources, nor does it equate to comprehensive facilitating conditions or institutional support; it also differs from teacher-level AI readiness, which centers on individual preparedness in terms of knowledge, skills, vision, and ethical considerations (Wang et al., 2023).

Research on teachers’ technology integration indicates that hardware, time, training, institutional support, and mismatches between curriculum and assessment jointly constrain classroom implementation (Ertmer, 1999; Hew and Brush, 2007). For art and design teachers, resources are only meaningful in practice when they can be embedded into open-ended tasks, process guidance, and work evaluation. Given the research focus, quantitative RR emphasizes only teachers’ perceptions regarding whether “relevant resources are accessible and suitable for their course”; training, institutional support, and governance norms are considered broader external contextual factors and will be examined separately during the qualitative phase.

Recent studies indicate that external contextual factors such as training, technical support, and transparent policies also influence teachers’ ability to transition AI from personal experimentation to formal instruction (Taheri et al., 2025; Zhao et al., 2025; Hazzan-Bishara et al., 2025). These factors were examined as potential contextual influences during the qualitative phase but were not included in the quantitative definition of the RR in this study. The quantitative RR focuses on resource availability and curriculum alignment, operationally excluding general attitudes toward AI, overall judgments of usefulness, and unconditional intentions to use AI. Given that some items in the original scale may cross boundaries, subsequent measurement validation and sensitivity analyses will separately assess their impact, rather than arbitrarily deleting items solely to improve model fit (Haynes et al., 1995).

### 2.4 Research model and hypotheses

Based on the above theory, this study retains the core relationships of TAM and incorporates RR as an external implementation condition into the model. All eight hypotheses were formulated prior to quantitative analysis based on existing theories and literature; the qualitative phase was used solely to interpret the patterns of quantitative relationships, not to alter the hypothesis directions *post hoc*.

Perceived Ease of Use (PEOU) refers to the extent to which an individual believes that using a particular technology requires little effort. According to the classic Technology Acceptance Model (TAM), lower learning and operational burdens enhance users’ perceptions of the technology’s performance usefulness, and meta-analyses on teachers’ technology adoption generally support the positive effect of PEOU on perceived usefulness (PU) (Davis, 1989; Scherer et al., 2019). In art and design education, teachers are more likely to recognize the pedagogical value of AI only when it can seamlessly integrate into studio activities such as concept development, visual exploration, feedback, and revision (Sawyer, 2017; Meyer and Norman, 2020). Therefore, we propose:

*H1*: Perceived ease of use (PEOU) positively influences perceived usefulness (PU).

Perceived usefulness (PU) refers to the extent to which teachers believe AI can improve their teaching performance. TAM and research on educational technology among teachers typically regard PU as a key cognitive antecedent of positive attitudes (Davis, 1989; Teo, 2011; Scherer et al., 2019). In art and design courses, if AI can support idea generation, visual exploration, feedback on student work, and classroom organization, teachers gain instrumental grounds for forming positive evaluations. While value concerns in creative education may limit the strength of this transformation, they do not alter the pre-established positive direction assumed in this study. Therefore, we propose:

*H2*: Perceived usefulness (PU) positively influences attitude (AT).

Behavioral intention (BI) refers to the extent to which teachers plan or are willing to continue using AI in teaching. The classic TAM model allows perceived usefulness (PU) to directly influence BI beyond attitudes, and research on teachers’ adoption of technology and generative AI also shows that recognition of the value of AI for teaching performance typically strengthens usage intentions (Davis et al., 1989; Teo, 2011; Kong et al., 2024). For art and design teachers, when AI is perceived as consistently supporting specific tasks such as lesson planning, creative inspiration, work evaluation, and feedback, they are more likely to form an intention to adopt it—even if other value judgments still require consideration. Therefore, we propose:

*H3*: Perceived usefulness (PU) positively influences behavioral intention (BI).

Attitude (AT) refers to teachers’ overall evaluation of using AI in teaching. According to TAM and teacher adoption research, the more positive the overall evaluation, the more likely it is to translate into usage intentions (Davis et al., 1989; Teo, 2011; Scherer et al., 2019). In art and design education, this evaluation also includes whether AI aligns with curricular goals, fosters originality, supports process training, and promotes professional responsibility. Only when teachers develop an overall acceptance in these areas are they more likely to plan to integrate AI into their instruction. Therefore, we propose:

*H4*: Attitude (AT) positively influences behavioral intention (BI).

Perceived ease of use is also considered a precursor to attitude in the classic TAM: lower learning costs and stronger perceived control over operations typically reduce frustration and promote positive evaluations (Davis et al., 1989; Teo, 2011; Scherer et al., 2019). The emphasis on creative processes and foundational skills in art and design education suggests that this relationship may be highly context-sensitive; however, prior literature does not yet provide sufficient evidence to assume a negative direction. To maintain comparability with the classic TAM and avoid revising hypotheses based on outcomes, this study retains the positive prior assumption:

*H5*: Perceived ease of use (PEOU) positively influences attitude (AT).

In addition to the classic TAM path, this study defines RR as teachers' perceptions of the availability and curriculum alignment of AI teaching resources, emphasizing whether these resources can support specific course planning and instructional implementation. This construct is related to facilitating conditions but does not include training, copyright guidance, or governance safeguards. Thus, RR may independently influence teachers' perceptions of ease of use, perceived usefulness, and attitudes.

First, PEOU focuses on the effort required for practical implementation. UTAUT and research on teachers' technology integration suggest that accessible infrastructure and task resources can reduce implementation barriers (Venkatesh et al., 2003; Ertmer, 1999; Hew and Brush, 2007). Art and design instruction relies on image-generating accounts, equipment, case resources, and teaching materials aligned with curricula; the easier these resources are to access and adapt to courses, the lower teachers perceive the cost of learning tools and organizing classroom activities. Therefore, it is proposed that:

*H6*: Resource readiness (RR) positively influences perceived ease of use (PEOU).

Second, PU focuses on whether AI can improve specific teaching tasks. External resources are more likely to have their performance value recognized by teachers only when they are accessible and aligned with the tasks and curriculum (Hazzan-Bishara et al., 2025; Zhao et al., 2025). In art and design courses, resources that can be directly applied to concept exploration, feedback on student work, and classroom demonstrations make the educational benefits of AI more visible. Therefore, it is proposed that:

*H7*: Resource readiness (RR) positively influences perceived usefulness (PU).

Finally, RR may also influence teachers’ overall attitudes toward AI in teaching. General technology acceptance research suggests that more favorable external implementation conditions can reduce uncertainty during the adoption process (Venkatesh et al., 2003; Teo, 2011). In art and design education, when teachers perceive that required AI teaching resources are accessible and adaptable to specific curricula, their uncertainty regarding implementation may decrease, thereby fostering a more positive overall attitude. Therefore, it is proposed that:

*H8*: Resource readiness (RR) positively influences attitude (AT).

**Figure 1:**
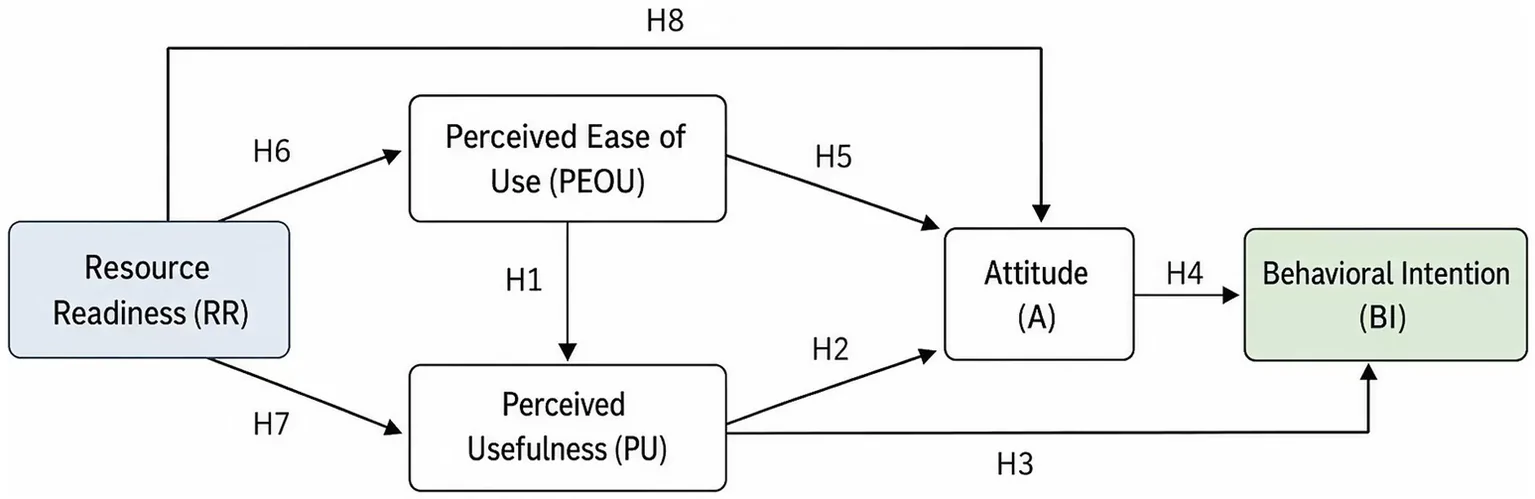
Conceptual model of art and design teachers’ intention to adopt AI in teaching.

In summary, this study’s model includes eight preset paths: PEOU → PU, PU → AT, PU → BI, AT → BI, PEOU → AT, RR → PEOU, RR → PU, and RR → AT. Figure 1 presents all relationships in the order of H1 to H8 as stated in the main text. All relevant information is presented in Figure 1.

## 3 Research methodology

### 3.1 Research design

This study employs an explanatory sequential mixed-methods design. The first phase examines the hypothesized relationships among resource readiness, perceived ease of use, perceived usefulness, attitude, and behavioral intention through questionnaires and structural equation modeling. The second phase provides contextual explanations for the supported, contradictory, and unstable estimates from the quantitative results via semi-structured interviews.

The quantitative and qualitative phases are sequentially connected in the order of QUAN→qual. The quantitative results first identify the relational patterns requiring further interpretation, which then guide the formulation of interview questions and thematic integration.

This design preserves the structured features of TAM relationship testing while allowing researchers to examine discipline-specific criteria unique to creative fields, such as originality, skill development, process authenticity, and pedagogical governance.

### 3.2 Participants and research context

The study focuses on art and design faculty from several universities in central China. The teaching environments of the surveyed instructors vary in terms of AI accounts, course resources, equipment, modular case studies, training opportunities, and institutional regulations, thus this study treats resource availability as a key contextual factor, rather than broadly characterizing the entire region as resource-poor.

A total of 101 valid questionnaires were collected during the quantitative phase, with no missing values for questions Q8–Q24. In the qualitative phase, eight respondents were purposefully selected from relevant teacher groups to cover visual communication, fashion design, and other art design disciplines, including teachers with varying degrees of AI exposure and differing stances on AI adoption. The interview sample was used to interpret patterns observed in the quantitative data, without aiming for statistical representativeness or making strong claims about theoretical saturation.

### 3.3 Quantitative research: survey procedures and measurement

#### 3.3.1 Survey procedure

The questionnaire was distributed online. Participants read the study instructions before beginning, and provided written informed consent after understanding the research purpose, anonymity principles, and voluntary participation requirements. The questionnaire collected demographic information as well as data on constructs such as perceived usefulness, perceived ease of use, resource readiness, attitudes, and behavioral intentions.

#### 3.3.2 Measurement instruments

All core items were measured using a five-point Likert scale (1 = strongly disagree, 5 = strongly agree). Perceived usefulness was assessed by Q8–Q10, perceived ease of use by Q11–Q13, resource readiness by Q14–Q17, attitude by Q18–Q20, and behavioral intention by Q21–Q24, totaling 17 items. The four RR items addressed the following: Q14 reflected willingness to use under conditions of available resources; Q15 concerned the pedagogical suitability of learning resource formats; Q16 focused on module clarity and feasibility of course scheduling; and Q17 evaluated content interest and attention maintenance. The definition of quantified RR was primarily reflected in Q15 and Q16; Q14 contained conditional behavioral intention components, while Q17 closely resembled an evaluation of teaching effectiveness. Based on the original research design, the main analysis retained Q14–Q17 to preserve traceability of the study design, without removing items *post hoc* to improve model fit. Sensitivity results were also reported after excluding Q14—the item with the most extreme boundary—using only Q15–Q17 to measure RR.

The correspondence between constructs and items is shown in Table 1.

**Table 1:** Correspondence between core constructs and items.

Construct	Abbreviation	Item range	Number of items
Perceived usefulness	PU	Q8–Q10	3
Perceived ease of use	PEOU	Q11–Q13	3
Resource readiness	RR	Q14–Q17	4
Attitude	AT	Q18–Q20	3
Behavioral intention	BI	Q21–Q24	4

#### 3.3.3 Quantitative analysis

The quantitative model was estimated using maximum likelihood estimation, with 5,000 case-level parameter bootstrap replications conducted to report bias-corrected 95% confidence intervals and bootstrap standard errors. Overall model fit was assessed using a Bollen–Stine bootstrap test with 2,000 replications (Bollen and Stine, 1992). Model fit was evaluated comprehensively based on χ^2^, degrees of freedom (df), χ^2^/df ratio, CFI, TLI, RMSEA and its 90% confidence interval, SRMR, AIC, and BIC. Given the sample size and inconsistencies observed between conventional ML tests and bootstrap results for certain paths, hypothesis testing primarily relied on whether the bootstrap confidence interval crossed zero.

The measurement model report includes standardized factor loadings, Cronbach’s *α*, composite reliability, AVE, latent variable correlation matrix, complete HTMT matrix, and 5,000 bootstrap confidence intervals (Henseler et al., 2015). To assess construct discriminant validity, we compared the original five-factor model, a four-factor model combining RR and AT, and a four-factor model combining AT and BI.

To assess the anomalous nature of the RR → AT estimate, we further examined the latent variable correlation between RR and AT, bootstrap standard errors and confidence intervals, variance inflation factors at the scale mean level, model residuals, and error/disturbance variances, and compared results from retaining all RR items, removing Q14, and excluding straight-line responders. To avoid model re-specification driven by results, this study did not post-hoc add residual correlations or modify preset paths based on modification indices. Sample size requirements for SEM depend on factor loadings, path coefficients, model complexity, and data distribution (Wolf et al., 2013; Muthén and Muthén, 2002); therefore, instead of claiming sample adequacy based on empirical rules such as “over 100,” we incorporated the *n* = 101 limitation into our interpretation.

### 3.4 Qualitative research: interview design and analysis

#### 3.4.1 Interview sampling

Purposeful sampling was used in the qualitative phase. The directional differences, unsupported relationships, and unstable estimates observed during the quantitative phase further guided the selection of interviewees and the focus of follow-up questions. These included the directional discrepancy between PEOU and AT, the unstable relationship between PU and AT, the relationship between RR and PEOU, as well as the risk of distinguishing RR from AT. Subsequently, teachers with varying levels of AI exposure and differing stances toward adoption were selected to obtain diverse interpretations. The verifiable characteristics of the eight participants are shown in Table 2.

**Table 2:** Overview of qualitative interview participants.

Code	Professional/Teaching focus	Institution information	AI engagement and overall stance
P1	Design major	A university in Hunan	Engaged with ChatGPT, Midjourney, etc.; overall positive, emphasizing redesign and copyright boundaries
P2	Visual Communication Design	A university in Hunan	Limited exposure to text-to-image tools; not yet used in the classroom, with a somewhat conditional attitude.
P3	Fashion Design	A university in Hunan	Limited AI exposure; recognize efficiency, yet concerned about taking shortcuts and diminished creativity
P4	Visual Communication Design	A university in Hunan	Engaged with Stable Diffusion and Midjourney; proactively explored their educational applications
P5	Fundamentals of Art and Design and Graphic Design	A university in Hunan	Engage with AI-powered drawing and writing; support using it as an aid, but oppose directly submitting generated results.
P6	Art and Design-related courses	A university in Hunan	Engaged with Midjourney and similar tools; inclined toward broad exploration of AI and curriculum evaluation reform
P7	Visual Communication and Digital Media	A university in Hunan	Study large models and GPT-related knowledge; advocate for selective and standardized usage.
P8	Visual Communication Design	A university in Hunan	Limited practical experience with AI; cautious attitude, willingness to use depends on school arrangements

The table reports only information that can be consistently verified from the anonymized aggregated data; no inferences were made as comparable data on gender, age, and years of teaching experience were not provided.

#### 3.4.2 Interview outline

The interview primarily revolved around the following questions:

1. How teachers understand the value and risks of AI in art and design education.
2. Why teachers do or do not formally incorporate AI into their courses.
3. How the ease of use of AI affects teachers’ judgments regarding originality, skill development, and the learning process.
4. What resources, support, and governance conditions influence teachers’ attitudes toward adoption and behavioral intentions.
5. Interview questions align with the quantitative model, yet allow respondents to raise professional concerns and counterexamples beyond the model. Quantitative results connect to the qualitative phase in two ways: first, by identifying the dimensions of variation needed for interview sampling, and second, by guiding follow-up questions around key structural relationships. Thus, interviews are not independent general opinion surveys, but rather targeted interpretations of the quantitative relationship patterns (Fetters et al., 2013).

All interview materials were originally conducted in Chinese. The available data include transcribed spoken texts and written Q&A records. Since the existing records do not consistently confirm whether all eight interviews were recorded, this study does not claim that all interviews were audio-recorded. Prior to analysis, direct identifiers such as names and specific affiliations were removed, and participants are referred to as P1–P8. Anonymized questionnaire data, interview transcripts, and related research records are stored and managed in accordance with Hunan Women’s University’s data protection policies. Analysis is based directly on verified Chinese texts without involving cross-linguistic translation.

#### 3.4.3 Qualitative analysis

The interview data were analyzed using thematic analysis, combining deductive and inductive coding. The analysis followed Braun and Clarke's (2006) six-phase procedure: familiarizing oneself with the data, generating initial codes, identifying candidate themes, reviewing themes against the full dataset, defining and naming themes, and selecting quotations that illustrate both common patterns and divergent views. Deductive coding began with RR, PEOU, PU, AT, and BI, while inductive coding was used to openly identify originality, skill weakening, taking shortcuts, process authenticity, copyright, academic integrity, and governance boundaries.

The research team reviewed key coding, thematic boundaries, and representative quotations; when discrepancies in understanding arose, they revisited the original context to compare coding definitions, discussed differences, and documented reasons for theme adjustments. The analytical process preserved the coding framework, records of theme revisions, counterexample checks, and analysis memos, creating a traceable audit trail. Qualitative findings were used to propose possible explanations for quantitative relationship patterns but were not considered causal evidence for statistical pathways.

To avoid retaining only supporting materials, the analysis simultaneously retrieved opposing views and counterexamples. Citations in the results section were preserved in their original meaning, with only repetitive filler words that do not affect the meaning removed, and obvious speech recognition errors corrected.

#### 3.4.4 Reliability and analytical adequacy

This study does not claim that the eight respondents achieved theoretical saturation, but instead seeks analytical adequacy appropriate to the research question through purposeful differential selection, cross-text thematic comparison, counterexample verification, and anonymized quotation of original texts.

Qualitative evidence serves to reveal how teachers understand professional value conflicts and external support conditions in AI-based teaching, complementing, corroborating, or contrasting with quantitative findings.

### 3.5 Integration of quantitative and qualitative results

Integration spans the three stages of connection, construction, and interpretation: first, quantitative results determine the focus of qualitative sampling and follow-up questions; second, in thematic analysis, evidence that aligns with, supplements, or contradicts structural pathways is preserved; finally, through joint presentation, hypotheses, qualitative themes, representative quotations, and counterexamples are systematically compared. Integration judgments distinguish between mutual reinforcement, complementary explanations, and discrepancies, without using qualitative data to re-evaluate whether hypotheses receive statistical support (Levitt et al., 2018; Fetters et al., 2013).

## 4 Research results

The model with the expanded sample received partial support. Bootstrap results supported H1, H3, H4, and H6; H2 and H7 had 95% confidence intervals crossing zero, thus not supported; H5 showed a significant negative relationship, contrary to the original positive hypothesis; although H8 was positive, it was not substantially interpreted due to its standardized coefficient exceeding 1 and insufficient discriminant validity between RR and AT.

### 4.1 Quantitative result

#### 4.1.1 Sample and data integrity

The quantitative analysis is based on 101 valid questionnaires. The research model includes five latent variables—PU, PEOU, RR, AT, and BI—comprising a total of 17 items. Items Q8–Q24 have no missing values.

Sample augmentation increased the information available for parameter estimation, but a sample size of 101 remains relatively small for a CB-SEM model involving five latent variables and eight structural paths. This study does not rely solely on fixed sample-size rules to assess adequacy, but instead considers fit indices, 5,000 bootstrap confidence intervals, competing measurement models, and item sensitivity results (Table 3).

**Table 3:** Sample and estimation information.

Project	Result
Quantitative valid sample	101
Missing values for Q8–Q24	0
Estimation methods	Maximum likelihood (ML)
Parameter bootstrap	5,000 replications; bias-corrected 95% CI
Bollen–Stine bootstrap	2,000 replications
Qualitative interviews	8 teachers

#### 4.1.2 Reliability and validity of the measurement model

In the original five-factor CFA, the standardized factor loadings for the 17 items ranged from 0.762–0.933. The Cronbach’s *α* for the five constructs ranged from 0.835–0.929, composite reliability from 0.859–0.934, and AVE from 0.643–0.781, indicating overall acceptable internal consistency and convergent validity. Complete results are presented in Appendix Table A1.

Discriminant validity still presents some local issues. In the five-factor CFA, the latent variable correlation between RR and AT was 0.949, and between AT and BI was 0.893; the corresponding HTMT values were 0.944 (95% percentile bootstrap CI [0.879, 1.003]) and 0.922 (95% percentile bootstrap CI [0.839, 0.987]). The former confidence interval includes 1, indicating that RR and AT are difficult to fully distinguish in this sample; the latter, although not including 1, has a point estimate exceeding 0.90, which should still be considered borderline evidence. Complete correlation and HTMT results are presented in Appendix Tables A2, A3.

The competing CFA results indicated that the original five-factor model (χ^2^(109) = 208.197, CFI = 0.937, TLI = 0.921, RMSEA = 0.095, SRMR = 0.059, AIC = 296.197, BIC = 411.262) outperformed both the RR–AT combined model and the AT–BI combined model. However, the RMSEA of the five-factor model remained relatively high, and the superior fit compared to the combined models does not resolve the issue of discriminant validity between RR and AT; thus, it should only be regarded as relative evidence supporting construct differentiation. See Appendix Table A4 for details.

Measurement quality and summary of key risks are shown in Table 4; complete measurement and results for the competing models are presented in Appendix Tables A1–A4.

**Table 4:** Summary of measurement quality and discriminant validity.

Indicator	Result	Explanation
Standardized factor loadings	0.762–0.933	Overall acceptable
Cronbach’s α	0.835–0.929	Good internal consistency
Composite reliability (CR)	0.859–0.934	Good composite reliability
AVE	0.643–0.781	Good convergent validity
HTMT: RR–AT	0.944 [0.879, 1.003]	Insufficient discriminant validity
HTMT: AT–BI	0.922 [0.839, 0.987]	Borderline evidence

#### 4.1.3 Structural model fit

The fit indices for the structural model were χ^2^(111) = 213.227, χ^2^/df = 1.921, CFI = 0.935, TLI = 0.920, SRMR = 0.059, and RMSEA = 0.096 (90% CI [0.076, 0.115]). The χ^2^/df, CFI, TLI, and SRMR indicate that the model meets some commonly used criteria for incremental and residual fit; however, the RMSEA remains above the typically recommended range. Therefore, the overall model fit is considered partially acceptable rather than good. A Bollen–Stine bootstrap test with 2,000 replications did not reject the model (*p* = 0.226), but this result does not eliminate the concern of approximate misfit suggested by the RMSEA. Given the sample size, issues regarding discriminant validity between RR and AT, and the unusual standardized estimate of the path from RR to AT, the structural path results should be regarded as tentative evidence requiring replication in an independent sample.

#### 4.1.4 Robustness and sensitivity analysis

All 5,000 parameter bootstrap replications produced valid results, with 4,999 reporting regular convergence indicators; no negative measurement error variances or negative latent variable disturbance variances occurred across all resamples. Given the sample size and inconsistencies observed in some path estimates between conventional ML tests and bootstrap results, path significance was primarily determined based on bias-corrected 95% confidence intervals from the 5,000 bootstrap replications.

The bootstrap standard error for H8 was 0.160, with a bias-corrected 95% confidence interval of [0.772, 1.417]. In the structural model, the latent variable correlation between RR–AT was 0.934, and the mean scale VIF values ranged from 2.02 to 2.35. No negative error variances or negative disturbance variances were detected; however, the highest residual correlation occurred between Q10 and Q14 (0.196), and Q14 showed positive residuals with several non-RR items, suggesting that item content overextension may still affect H8. Removing Q14 improved fit indices (CFI = 0.955, RMSEA = 0.083), but the HTMT for RR–AT remained at 0.926, and H8 remained at 0.978. Results were similar after excluding seven straight-line responders. Therefore, sensitivity analyses did not resolve the construct overlap issue, and the main analysis retained the original items without confirmatory interpretation of H8. See Appendix Table A5 for details.

#### 4.1.5 Hypothesis testing and structural relationships

Given the inconsistency between conventional ML tests and bootstrap results for H2 and H7, this study primarily relies on bias-corrected 95% confidence intervals based on 5,000 bootstrap replications as the main criterion, while also reporting bootstrap standard errors. The results are shown in Table 5.

**Table 5:** Results of bootstrap hypothesis tests.

Hypothesis	Path	Standardized *β*	Bootstrap SE	Bias-corrected 95% CI	Conclusion
H1	PEOU → PU	0.548	0.176	[0.084, 0.810]	Supported
H2	PU → AT	0.311	0.190	[−0.066, 0.682]	Not supported
H3	PU → BI	0.311	0.133	[0.032, 0.557]	Supported
H4	AT → BI	0.646	0.137	[0.375, 0.917]	Supported
H5	PEOU → AT	−0.389	0.182	[−0.832, −0.101]	Not supported; opposite direction
H6	RR → PEOU	0.761	0.087	[0.566, 0.902]	Supported
H7	RR → PU	0.306	0.186	[−0.023, 0.722]	Not supported
H8	RR → AT	1.005	0.160	[0.772, 1.417]	Not supported; anomalous estimate

*H1 is supported*: PEOU significantly and positively influences PU (*β* = 0.548, bias-corrected 95% CI [0.084, 0.810]). This indicates that the more teachers perceive AI tools as easy to understand and use, the more likely they are to recognize their educational value.

*H3 is supported*: PU significantly and positively influences BI (*β* = 0.311, bias-corrected 95% CI [0.032, 0.557]). Perceived usefulness was directly and positively associated with teachers’ behavioral intention. Because PU → AT was not supported by the bootstrap results, this study does not claim a stable indirect effect of perceived usefulness through attitude.

*H4 is supported*: AT significantly and positively influences BI (*β* = 0.646, bias-corrected 95% CI [0.375, 0.917]). The point estimate of the standardized AT → BI coefficient was higher than that of PU → BI; however, the two paths were not formally compared, so this difference is reported descriptively only. In addition, the relatively high HTMT between AT and BI warrants caution in interpreting the magnitude of this association.

*H6 is supported*: RR significantly and positively influences PEOU (*β* = 0.761, bias-corrected 95% CI [0.566, 0.902]). The more teachers perceive AI teaching resources as accessible and adaptable to their courses, the more likely they are to believe that AI teaching can be practically implemented and integrated into instruction.

H2 and H7 were not supported. The *β* for PU → AT was 0.311, with a bias-corrected 95% CI [−0.066, 0.682]; the *β* for RR → PU was 0.306, with a 95% CI [−0.023, 0.722], both intervals crossing zero. The estimate for H5 was negative, with a confidence interval that did not cross zero (*β* = −0.389, 95% CI [−0.832, −0.101]), thus rejecting the original positive hypothesis and indicating an inverse relationship. H8 showed a positive effect (*β* = 1.005, 95% CI [0.772, 1.417]), but the standardized coefficient exceeded 1, and the discriminant validity between RR and AT was insufficient; therefore, it is not considered reliable support.

Overall, H1, H3, H4, and H6 were supported; H2, H5, and H7 were not supported, with H5 pointing in the opposite direction of the hypothesis; H8 was not evaluated for support due to estimation anomalies and issues with discriminant validity.

### 4.2 Qualitative results

Qualitative analysis was used to understand the support, lack of support, directional differences, and measurement risks presented by H1–H8. Table 6 shows the correspondence between the five themes, representative quotations, and all eight structural relationships.

**Table 6:** Joint display of quantitative and qualitative results.

Quantitative results	Qualitative themes and evidence	Integrated explanation
H1 supported	AI reduces operational burden and improves material/feedback efficiency (P1, P4)	Ease of use helps teachers identify specific educational value.
H2 not supported	Useful but not value-neutral (P2, P8)	Functional value alone is insufficient to form a positive attitude.
H3 supported	Teachers are willing to conditionally use AI in specific tasks (P1, P5)	Specific teaching effectiveness can be directly linked to the intention to use AI.
H4 supported	Overall attitude includes governance, copyright, and responsibility assessment (P4, P6)	A more favorable overall attitude is associated with stronger behavioral intention.
H5 opposite to the hypothesized direction	Low barriers are also understood as risks of taking shortcuts and weakening skills (P3, P8).	Qualitative evidence provides possible explanations but does not prove causation.
H6 supported; H8 anomalous	Account, device, and course case correspond to RR; training and institutional support are external contextual factors identified in the interviews (P4, P8).	Resource enhancement can improve implementation feasibility, but there remains a risk of conceptual overlap between RR and AT.
H7 not supported	Resources being in place first address the question of “whether it can be implemented,” without automatically answering “whether it has educational value.”	Distinguishing between resource availability and usefulness assessment

#### 4.2.1 Theme 1: artificial intelligence has practical value in teaching but is not a value-neutral teaching tool

The interview first revealed that most teachers do not deny the educational value of artificial intelligence. Teachers generally believe that AI can improve lesson preparation efficiency, accelerate information gathering, enrich classroom teaching formats, and provide inspirational support when students’ creativity is blocked. Some teachers noted that in the past, finding suitable images and case studies required considerable time, but now simply entering keywords allows them to quickly generate a large amount of usable materials. Others pointed out that AI helps broaden students’ thinking, enhances classroom vitality, and makes student work more diverse and vibrant.

However, these positive evaluations do not mean that teachers view artificial intelligence as a “value-neutral” tool. In interviews, teachers repeatedly emphasized that AI should only serve as an auxiliary and reference aid, not replace core training or the final creative outcomes. Even if AI can enhance efficiency, teachers still question whether it alters teaching objectives, learning processes, and criteria for evaluating student work. Thus, in art and design education, the usefulness of AI is not an automatic technological attribute that leads to positive attitudes, but rather a pedagogical resource that holds practical value yet requires careful handling. This perspective helps explain why teachers conceptually accept PU, yet the direct path from PU to AT fails to form a stable and significant connection within the structural model.

For example, P1 stated: “By entering keywords, the process of creating courseware materials can greatly improve efficiency and expand content possibilities.” However, P2 emphasized: “AI is merely a tool; it’s not all-powerful. Some details still require designers to refine with esthetic judgment.” These two perspectives together indicate that efficiency evaluation does not entirely equate to overall acceptance.

#### 4.2.2 Theme 2: ease of use as a double-edged sword in creative teaching

The interview revealed a dual understanding among teachers regarding “ease of use.” On one hand, teachers acknowledged that AI can quickly generate images, reduce repetitive tasks, and lower the threshold for certain operations. Thus, ease of use can enhance teachers’ perception of AI’s practical functions, aligning with the positive relationship between perceived ease of use (PEOU) and perceived usefulness (PU).

On the other hand, in creative teaching, teachers do not always regard “convenience” as an unconditional advantage. A low threshold may be interpreted as a warning sign that students skip thinking, trial and error, reflection, and revision, turning learning into a process of “quick generation—direct use.” These narratives provide a contextual explanation for the directional differences between PEOU and AT, but they do not prove that ease of use leads to negative attitudes.

P8 expressed concern that students, after using AI, “might neglect learning certain professional knowledge and become lazy”; P3 similarly summarized this risk as “the most common skill might take shortcuts.” These quotes illustrate why teachers may perceive low barriers as both a reduction in teaching costs and an increase in process-related risks.

#### 4.2.3 Theme 3: originality, deskilling, and the shortcut problem

The most consistent and prominent focus during the interviews centered on originality, foundational skill training, and the issue of “taking shortcuts.” Several teachers pointed out that artificial intelligence can be used to assist in sketching, ideation, or inspiration generation, but cannot directly replace final works or professional expression. Teachers generally emphasized that students must still maintain original thinking and creative abilities; otherwise, even if their work is rich in form, it may lack depth of creativity.

Meanwhile, concerns about deskilling are particularly prominent. Some teachers worry that when students become overly reliant on artificial intelligence, they may gradually lose the ability to think independently, express themselves through handwriting, make visual judgments, and engage in careful deliberation, developing instead a habit of “letting AI generate content first, then making minor edits.” As such, the more convenient and user-friendly AI becomes, the more likely teachers are to be cautious about its erosion of originality and professional training. This further explains why perceived usefulness (PU) and perceived ease of use (PEOU) cannot directly and consistently translate into positive attitudes: teachers’ attitudes are not automatically determined by technological performance, but are filtered and shaped by the core values of creative disciplines.

P4 stated: “I only allow the use of AI technology during the sketch and conceptual phase for design proposals, but the final product must be an original creation with high personal authorship.” P7 was even more direct: “If you treat it purely as a ghostwriter, then this graduation project has little to do with you.” This indicates that the interviewed teachers generally support limited auxiliary use of AI, while opposing its replacement of students’ core creative expression.

#### 4.2.4 Theme 4: governance and academic integrity as prerequisites for developing a positive attitude

The interview also revealed that teachers’ attitudes toward artificial intelligence depend not only on its teaching effectiveness but are also influenced by governance standards and the state of academic integrity. Some teachers explicitly pointed out that AI-generated images and texts could lead to copyright disputes, unclear authorship, distorted evaluations, and risks to academic integrity. A number of teachers believe that if schools continue using outdated grading criteria for assignments in response to AI-assisted learning, students may become overly reliant on AI to “get by” with their work, making it difficult for teachers to fairly assess students’ actual capabilities.

This theme aligns with the positive relationship between AT and BI. Here, “attitude” does not simply refer to liking or disliking, but rather represents teachers’ comprehensive judgment about whether AI possesses pedagogical controllability—whether it has boundaries, is explainable, and can be integrated into course assessment and academic standards. Only after forming a relatively positive overall evaluation are teachers more likely to express clear intentions to adopt it.

P6 summarized the basic boundary as: “AI should still be used as a tool, not as a brain.” P4 pointed out that some generative models may imitate and reassemble existing artists’ works, “so there is to some extent a copyright risk involved in the patterns.” These comments indicate that teachers’ overall attitude includes considerations regarding responsibility, copyright, and traceability of evaluation.

#### 4.2.5 Theme 5: resource preparation as a prerequisite for adoption

Finally, the interviews clearly indicated that resource availability is not a secondary factor, but rather a prerequisite for teachers’ willingness to actually adopt artificial intelligence. Several teachers mentioned that they would be more inclined to learn and use AI if schools could provide accounts, databases, high-performance computers, software subscriptions, open resource libraries, clear case studies, and modular support. Without updated teaching materials, coordinated school arrangements, and accessible resources, even when teachers recognize the potential of AI, they often do not proactively integrate it into formal instruction.

This narrative of “first acquiring resources, then implementing instruction” aligns with the positive relationship between RR and PEOU: access to accounts, devices, databases, and course examples first influences whether teachers perceive AI as easy to control and integrate into their teaching. Interviews also revealed broader external contextual factors such as training, institutional arrangements, and school openness; these factors are not directly measured by the RR scale and thus are not used to expand the quantitative meaning of RR. Some teachers linked resource availability and institutional support to their overall attitudes, suggesting potential overlapping contextual sources between RR and AT measures, but this does not constitute independent causal evidence that RR directly affects AT.

P4 stated: “It would be ideal if schools could provide relevant funding and create conditions for teachers to learn and research AI technologies.” P8 also suggested that if schools could offer resource libraries and software, teachers would adjust their teaching according to unified arrangements. Both statements link resource support with feasibility.

Differences of opinion also emerged during the interviews. P6 argued that AI could be integrated into “all disciplines, even thinking training courses,” and believed that course evaluation itself should adapt to AI involvement; in contrast, P8 stated that AI is currently “not needed” and whether it will be adopted in the future depends mainly on school arrangements. The analysis preserves this distinction between proactive exploration and cautious observation, without generalizing the eight teachers into a single attitude.

#### 4.2.6 Integration and interpretation of quantitative and qualitative research findings

Quantitative and qualitative results together present a more contextualized picture. H1, H3, H4, and H6 are supported, indicating that ease of use enhances perceived usefulness, both usefulness and attitude are related to behavioral intention, and resource readiness primarily influences teachers’ judgments about the ease of implementing AI.

H2 was not supported, while H5 showed a significant negative relationship. Interviews indicated that teachers do not automatically equate functionality and convenience with educational legitimacy; they also assess whether low barriers might encourage students to take shortcuts, undermine originality and skill development, or blur the boundaries of authorship responsibility, course assessment, and academic integrity. This qualitative evidence offers possible explanations for the inverse relationship but does not establish causal mechanisms.

H8 must be handled with caution. The standardized coefficient from RR to AT exceeds 1, and the HTMT between RR and AT is 0.944 (95% CI [0.879, 1.003]), indicating insufficient discriminant validity in the current measurement. In interviews, teachers frequently linked resource conditions with overall attitudes, and also separately mentioned institutional arrangements and school openness; the latter two are external contextual factors identified during the qualitative phase, rather than indicators of the RR scale.

Therefore, this study regards RR as an important external implementation condition, but does not interpret H8 as a reliable direct effect of resource readiness on attitude.

Overall, some core relationships in TAM received support, but the attitudes of art and design teachers were not linearly determined by perceived usefulness and ease of use. The inverted relationship for H5 suggests that technological convenience may simultaneously imply both efficiency and teaching risks; this conclusion still needs to be verified in future studies with clearer discriminant validity and larger samples.

## 5 Discussion

After expanding the sample and conducting supplementary sensitivity analyses, the research model still received only partial support. The paths PEOU → PU, PU → BI, AT → BI, and RR → PEOU were supported; however, PU → AT and RR → PU were not supported by bootstrap analysis. A significant negative relationship was observed between PEOU and AT, while the path RR → AT could not be reliably interpreted due to an anomalous coefficient and insufficient discriminant validity. The Bollen–Stine bootstrap test did not reject the model, but RMSEA remained relatively high, and the overlap between RR and AT persisted across item and sample sensitivity analyses, thus conclusions should be cautiously drawn.

### 5.1 Usefulness and attitude jointly influence behavioral intention

Both H3 and H4 were supported, indicating that perceived usefulness and attitude are directly and positively related to teachers’ intention to use AI in teaching. Among them, the coefficient for AT → BI is relatively large, suggesting that teachers’ judgments regarding the overall acceptability and legitimacy of AI teaching are closely associated with their intention to continue using it.

The interview revealed that this attitude involves not only a preference for or against the tools, but also a comprehensive judgment regarding course objectives, originality, skill development, fairness in assessment, and ethical boundaries. Therefore, even if AI is considered useful, teachers may still maintain a conditional acceptance overall.

In practice, promoting AI in education requires not only demonstrating its specific teaching value but also addressing teachers’ judgments regarding curriculum alignment and governance boundaries.

### 5.2 Usefulness has not consistently translated into attitudes

The support for H3 but not for H2 indicates that perceived usefulness can directly influence behavioral intention, yet may not consistently translate into overall attitude. In other words, teachers might intend to use AI in specific tasks while still remaining skeptical about its educational legitimacy.

Qualitative findings offer a possible explanation: AI holds practical value in lesson preparation, information retrieval, classroom presentations, and creative inspiration; however, teachers still question whether this efficiency alters learning tasks, undermines process training, or challenges the criteria for evaluating student work.

Therefore, the lack of support for PU → AT cannot be interpreted as teachers denying the use of AI, but rather suggests that usefulness is merely one necessary consideration in attitude formation, not a sufficient condition.

### 5.3 Ease of use and attitude are inversely related

The standardized coefficient for H5 is −0.389, with a bias-corrected 95% confidence interval of [−0.832, −0.101], indicating that the original positive hypothesis is not supported and suggesting an inverse relationship instead. Given that the study used cross-sectional self-report data, this result can only be interpreted as a conditional association and does not directly imply that ease of use causes negative attitudes.

In the interview, teachers acknowledged that AI-generated content is fast and easy to use, but expressed concern that its speed and simplicity might lead students to skip over brainstorming, trial and error, reflection, and revision, thereby reducing creative learning to merely inputting prompts and selecting outputs.

This finding suggests that ease of use in creative disciplines may be a double-edged signal: it can enhance perceived usefulness, yet also trigger teachers’ concerns about originality, skill dilution, and authenticity of the process. Future research needs to test this potential mechanism through longitudinal or experimental designs.

### 5.4 Resource readiness is primarily linked to implementation feasibility

The fact that H6 was supported while H7 was not indicates a relatively stable relationship between resource readiness and perceived ease of use, but this does not consistently enhance perceived usefulness.

The RR referred to in the quantitative results primarily concerns the availability and course compatibility of resources such as accounts, databases, devices, and course cases. These conditions first reduce implementation costs, making AI easier to integrate into actual teaching processes. Interviews additionally highlighted the importance of training support and clear institutional boundaries, but these two factors are not included in the measurement scope of this study’s RR scale.

Therefore, resource preparation primarily addresses whether implementation can proceed smoothly, rather than automatically answering whether it has educational value.

This result supports interpreting RR as external implementation conditions, but it does not justify claiming that RR directly determines teachers’ attitudes or judgments of usefulness.

### 5.5 Anomalous estimation of the RR–AT relationship

H8 has a *β* of 1.005, a bootstrap standard error of 0.160, and latent variable correlations between RR and AT, as well as HTMT values, of 0.949 and 0.944, respectively. The VIF at the scale mean level does not indicate severe multicollinearity, and the model shows no negative error or disturbance variances. However, the simultaneous occurrence of standardized coefficients exceeding 1 and insufficient discriminant validity still suggests potential construct overlap at the latent variable level or an unstable model specification.

The content review of the RR items further illustrates this risk: Q14 directly involves willingness to use under resource availability conditions, and Q17 includes evaluations of outcomes related to interest and attention maintenance, while only Q15 and Q16 focus more on resource format and course implementation arrangements. Although model fit improves after removing Q14, the HTMT of RR–AT remains at 0.926, and H8 is still close to 1, indicating that the issue cannot be attributed to a single item, nor can the scale be reconstructed *post hoc* based on this finding.

Based on this, the study retains the original RR measurement to maintain traceability of the research design, does not consider H8 as supported, and explicitly acknowledges the difficulty in empirically distinguishing between RR and AT within the current sample as a clear limitation. One possible explanation is that some respondents did not clearly distinguish between whether AI teaching resources were sufficiently prepared and whether they personally endorsed adopting AI for teaching. In particular, Q14’s conditional use-intention component and Q17’s judgment of teaching outcomes may have allowed attitudinal evaluations to enter the resource-readiness measure. Future studies should revise RR item wording through cognitive interviews and pretesting, measure resource availability, curriculum alignment, and overall attitude separately, compare alternative operationalizations based on specific resource inventories or facilitating conditions, and validate construct separation in independent samples.

### 5.6 Theoretical significance

This study presents three cautious theoretical implications.

First, the support for PEOU → PU, PU → BI, and AT → BI indicates that TAM still holds explanatory power; however, the lack of support for PU → AT and the inverse relationship between PEOU → AT suggest that attitude formation in creative teaching may be influenced by subject-specific value judgments.

Second, the interview results suggest a possible value filtering process: teachers re-evaluate the convenience and functionality of AI through criteria such as originality, foundational skills, authenticity of process, governance, and academic integrity. This process is currently an initial interpretation formed through mixed-method integration, rather than a causally validated mechanism.

Third, the support for RR → PEOU indicates that resource conditions primarily influence perceived implementation feasibility; however, the anomalous estimation of RR → AT suggests that the conceptual boundaries of resource readiness still require further clarification.

### 5.7 Practical significance

This study also has several practical implications for the promotion of artificial intelligence in art and design education.

First, schools and administrators should not simply demand teachers to adopt artificial intelligence based solely on its efficiency advantages. Research shows that “usefulness” does not automatically equate to “willingness to accept,” so policies and training should go beyond mere functional demonstrations and instead address teachers’ core concerns regarding originality, foundational skill development, authenticity of the learning process, and academic integrity. In other words, the promotion of artificial intelligence should not be limited merely to tool dissemination, but should focus on co-constructing both teaching value and usage boundaries.

Second, AI training should shift from merely teaching teachers how to operate AI toward helping them use it appropriately in creative education. For art and design teachers, the priority is not only to learn prompting techniques, but also to understand how AI can be positioned as an assistive, referential, and inspirational tool without replacing students’ core creative processes. Training should address how to design AI-supported learning tasks that preserve originality requirements, adapt assessment to identify students’ actual capabilities, and establish clear boundaries for AI use in courses.

Third, resource availability and curriculum alignment are crucial prerequisites for promoting application. Schools can prioritize providing account access, databases, software subscriptions, equipment, and modular teaching cases to reduce teachers’ implementation costs in integrating AI into their courses. Interviews also highlighted the importance of institutionalized training; however, this factor is an external contextual condition identified during the qualitative phase and is not directly measured by the RR scale.

Finally, schools should establish clearer governance and integrity frameworks. Teachers are cautious about artificial intelligence—not entirely opposed to the technology—but concerned about the lack of clear boundaries regarding copyright, assessment, fairness, and academic integrity. Therefore, promoting AI in teaching must be accompanied by updates to course policies, assignment requirements, authorship guidelines, and evaluation mechanisms, to strengthen teachers’ confidence in maintaining control over AI use in education.

### 5.8 Limitations and future research

This study still has notable limitations. First, a sample size of *n* = 101 remains relatively small for a CB-SEM model involving five latent variables and eight pathways. Although the study has increased the number of parameter bootstrap replications to 5,000 and conducted a Bollen–Stine bootstrap test, competing CFA, and sample/item sensitivity analyses, these procedures cannot replace independent replication with a larger sample, nor do they confirm adequate sample size; future research should perform model-specific Monte Carlo power analyses based on expected loadings, paths, and distributions. Second, the RMSEA value of 0.096 indicates only partially acceptable overall model fit.

Third, the HTMT values for RR–AT and AT–BI were 0.944 and 0.922, respectively, indicating that discriminant validity has not yet been sufficiently established; H8 showed an anomalous estimate with *β* > 1. The RR items encompass conditional intention, resource fit, module arrangement, and teaching outcomes, and respondents may not have clearly distinguished judgments of resource readiness from their overall attitudes toward AI adoption. Although the main analysis retained the original items and reported item-deletion sensitivity, this did not resolve construct overlap. Future research should revise RR item wording through cognitive interviews and pretesting, or compare alternative operationalizations based on concrete resource-availability inventories, curriculum-alignment indicators, and facilitating conditions, and then retest the measurement model in an independent sample. Accordingly, interpretations of the magnitudes of RR → AT and AT → BI should be considered exploratory.

This study focuses on specific regions and specialized teachers, and its findings are clearly context-dependent. Future research could compare different regions, institutional levels, and creative disciplines to examine whether the inverse relationship remains stable.

The qualitative sample consisted of only eight participants, primarily intended to provide clues for mechanism explanations. The existing anonymous materials support analyses of professional background, differences in AI exposure, verbatim quotations, and counterexample reporting, but do not offer consistent demographic information for comparison. Future research should employ standardized interview logs, combined with classroom observations, process logs, and longitudinal tracking, to enhance procedural transparency and the credibility of interpretations.

### 5.9 Discussion summary

This study did not fully validate the extended TAM model, but instead found that some classical pathways received support, while also revealing a reverse relationship between PEOU and AT, as well as measurement overlap between RR and AT. The results suggest preliminarily that AI adoption among creative subject teachers is influenced not only by instrumental beliefs but also jointly shaped by originality, skill development, process authenticity, and governance conditions.

## 6 Conclusion

Based on 101 questionnaires and interviews with 8 teachers, this study found support for the paths PEOU → PU, PU → BI, AT → BI, and RR → PEOU; however, PU → AT and RR → PU were not supported by bootstrap analysis. A significant negative relationship was observed between PEOU and AT, while the path RR → AT could not be reliably interpreted due to an anomalous coefficient and issues with discriminant validity.

These findings suggest that AI’s ease of use can enhance perceived usefulness, but in art and design education, convenience does not necessarily lead to positive attitudes. Teachers also assess whether AI undermines originality, foundational skills, trial and error, and authorship responsibility, and may thus remain cautious about excessive ease of use.

Theoretically, this study provides preliminary evidence for the contextual application of TAM in creative teaching, rather than establishing a stable boundary mechanism. Practically, quantitative findings highlight the importance of resource availability and curriculum fit, while interviews further suggest external contextual factors such as training and governance rules. The scope of these two types of evidence should not be conflated.

Given the limited sample size, only partially acceptable model fit, and insufficient discriminant validity between RR and AT as well as between AT and BI, the findings of this study should not be generalized to broader populations of teachers. Although 5,000 bootstrap replications and multiple sensitivity analyses enhanced transparency of results, they did not eliminate measurement overlap; future research should revalidate the measurement model and structural paths in an independent, larger sample.

Overall, the adoption of AI by art and design teachers is not merely an efficiency-driven decision, but a judgment process shaped by instrumental beliefs, professional values, teaching objectives, ethical governance, and resource conditions.

## Data Availability

The anonymized data supporting the conclusions of this article are available from the corresponding author upon reasonable request, subject to participant informed consent, institutional ethical requirements, and applicable data protection requirements. Potentially identifiable information and non-anonymized interview materials will not be made publicly available.

## Supplementary material

Supplementary Material 1 Appendix A: Supplementary results of quantitative sensitivity.
