# Supplementary material for: Understanding art and design teachers’ willingness to adopt artificial intelligence in teaching under resource constraints: a mixed-methods study on perceived usefulness, resource readiness, and creativity-related concerns
Source: Front Psychol. 2026 Sep 3;17:1854412. doi: 10.3389/fpsyg.2026.1854412 (PMC13582513; doi:10.3389/fpsyg.2026.1854412)
Supplement: Supplementary file 1 [file Table_1.docx]

**Appendix A. Supplementary Results of Quantitative Sensitivity Analysis**

The following supplementary tables present the complete results for the measurement model, discriminant validity, competing CFA models, and RR sensitivity analyses.

All main conclusions remain based on the original five-factor structure model retaining Q14–Q17.

**Appendix Table A1. Standardized Factor Loadings and Construct Reliability**

| **Construct** | **Item** | **Standardized load** | **Cronbach's α** | **CR** | **AVE** |
| --- | --- | --- | --- | --- | --- |
| PU | Q8 | 0.781 | 0.835 | 0.859 | 0.671 |
| PU | Q9 | 0.800 |  |  |  |
| PU | Q10 | 0.873 |  |  |  |
| PEOU | Q11 | 0.864 | 0.907 | 0.907 | 0.765 |
| PEOU | Q12 | 0.894 |  |  |  |
| PEOU | Q13 | 0.865 |  |  |  |
| RR | Q14 | 0.762 | 0.879 | 0.878 | 0.643 |
| RR | Q15 | 0.785 |  |  |  |
| RR | Q16 | 0.875 |  |  |  |
| RR | Q17 | 0.779 |  |  |  |
| AT | Q18 | 0.889 | 0.907 | 0.909 | 0.770 |
| AT | Q19 | 0.883 |  |  |  |
| AT | Q20 | 0.859 |  |  |  |
| BI | Q21 | 0.821 | 0.929 | 0.934 | 0.781 |
| BI | Q22 | 0.932 |  |  |  |
| BI | Q23 | 0.933 |  |  |  |
| BI | Q24 | 0.842 |  |  |  |

Note: Loadings are from the original five-factor CFA; α, CR, and AVE are shown only in the first row of each construct.

**Appendix Table A2. Five-Factor CFA Latent Variable Correlation Matrix**

| **Construct** | **PU** | **PEOU** | **RR** | **AT** | **BI** |
| --- | --- | --- | --- | --- | --- |
| PU | 1.000 |  |  |  |  |
| PEOU | 0.784 | 1.000 |  |  |  |
| RR | 0.730 | 0.760 | 1.000 |  |  |
| AT | 0.736 | 0.626 | 0.949 | 1.000 |  |
| BI | 0.784 | 0.625 | 0.786 | 0.893 | 1.000 |

Note: Report latent variable correlation coefficients; RR–AT correlation is 0.949.

**Appendix Table A3. Full HTMT and 5,000 Bootstrap Confidence Intervals**

| **Construct validity** | **HTMT** | **Bootstrap percentile 95% CI** | **Judgment** |
| --- | --- | --- | --- |
| PU–PEOU | 0.787 | [0.662, 0.892] | Not exceeding 0.90 |
| PU–RR | 0.741 | [0.585, 0.871] | Not exceeding 0.90 |
| PU–AT | 0.733 | [0.598, 0.849] | Not exceeding 0.90 |
| PU–BI | 0.770 | [0.664, 0.870] | Not exceeding 0.90 |
| PEOU–RR | 0.754 | [0.578, 0.895] | Not exceeding 0.90 |
| PEOU–AT | 0.631 | [0.452, 0.788] | Not exceeding 0.90 |
| PEOU–BI | 0.642 | [0.509, 0.765] | Not exceeding 0.90 |
| RR–AT | 0.944 | [0.879, 1.003] | Insufficient discriminant validity |
| RR–BI | 0.824 | [0.717, 0.915] | The point estimate did not exceed 0.90 |
| AT–BI | 0.922 | [0.839, 0.987] | Borderline evidence |

Note: The confidence interval is the percentile interval based on 5,000 Bootstrap samples; HTMT is not used as the sole criterion and should be interpreted in conjunction with competitive CFA, latent variable correlations, and item content.

**Appendix Table A4. Comparison of Competitive Measurement Models**

| **Model** | **χ²** | **df** | **χ²/df** | **CFI** | **TLI** | **RMSEA** | **SRMR** | **AIC** | **BIC** |
| --- | --- | --- | --- | --- | --- | --- | --- | --- | --- |
| Five-factor model | 208.197 | 109 | 1.910 | 0.937 | 0.921 | 0.095 | 0.059 | 296.197 | 411.262 |
| RR+AT combination | 235.358 | 113 | 2.083 | 0.922 | 0.907 | 0.104 | 0.061 | 315.358 | 419.963 |
| AT+BI merge | 262.350 | 113 | 2.322 | 0.905 | 0.886 | 0.115 | 0.057 | 342.350 | 446.955 |

Note: The three competing CFA models use the same 17 observed items, allowing for comparison of AIC and BIC; lower values indicate relatively better fit.

**Appendix Table A5. RR Items and Data Quality Sensitivity Analysis**

| **Analysis** | **n** | **CFI** | **TLI** | **RMSEA** | **SRMR** | **RR–AT HTMT** | **H8 β** |
| --- | --- | --- | --- | --- | --- | --- | --- |
| Main model: RR = Q14–Q17 | 101 | 0.935 | 0.920 | 0.096 | 0.059 | 0.944 | 1.005 |
| Remove Q14: RR = Q15–Q17 | 101 | 0.955 | 0.943 | 0.083 | 0.053 | 0.926 | 0.978 |
| Exclude 7 strictly linear responders | 94 | 0.939 | 0.925 | 0.092 | 0.060 | 0.943 | 1.024 |

Note: The model without Q14 has one fewer observed item, so its χ², AIC, and BIC are not directly compared with the main model in terms of goodness-of-fit. Although this model improves some fit indices, the HTMT for RR–AT still exceeds 0.90, and H8 remains close to 1; therefore, it does not replace the main model. Strict straight answering refers to all items from Q8–Q24 having identical response values.
